# Autophagy inducer rapamycin treatment reduces IFN-I–mediated Inflammation and improves anti–HIV-1 T cell response in vivo

**DOI:** 10.1172/jci.insight.159136

**Published:** 2022-11-22

**Authors:** Wenli Mu, Valerie Rezek, Heather Martin, Mayra A. Carrillo, Shallu Tomer, Philip Hamid, Miguel A. Lizarraga, Tristan D. Tibbe, Otto O. Yang, Beth D. Jamieson, Scott G. Kitchen, Anjie Zhen

**Affiliations:** 1 Division of Hematology/Oncology, Department of Medicine and; 2 UCLA AIDS Institute and the Eli and Edythe Broad Center of Regenerative Medicine and Stem Cell Research, David Geffen School of Medicine at UCLA, Los Angeles, California, USA.; 3 Statistic Core, Department of Medicine at UCLA, Los Angeles, California, USA.; 4 Division of Infectious Disease and; 5 Department of Microbiology, Immunology, and Molecular Genetics, David Geffen School of Medicine, UCLA, Los Angeles, California, USA.

**Keywords:** AIDS/HIV, Inflammation, Cellular immune response, Innate immunity

## Abstract

A hallmark of HIV-1 infection is chronic inflammation, even in patients treated with antiretroviral therapy (ART). Chronic inflammation drives HIV-1 pathogenesis, leading to loss of CD4^+^ T cells and exhaustion of antiviral immunity. Therefore, strategies to safely reduce systematic inflammation are needed to halt disease progression and restore defective immune responses. Autophagy is a cellular mechanism for disposal of damaged organelles and elimination of intracellular pathogens. Autophagy is pivotal for energy homeostasis and plays critical roles in regulating immunity. However, how it regulates inflammation and antiviral T cell responses during HIV infection is unclear. Here, we demonstrate that autophagy is directly linked to IFN-I signaling, which is a key driver of immune activation and T cell exhaustion during chronic HIV infection. Impairment of autophagy leads to spontaneous IFN-I signaling, and autophagy induction reduces IFN-I signaling in monocytic cells. Importantly, in HIV-1–infected humanized mice, autophagy inducer rapamycin treatment significantly reduced persistent IFN-I–mediated inflammation and improved antiviral T cell responses. Cotreatment of rapamycin with ART led to significantly reduced viral rebound after ART withdrawal. Taken together, our data suggest that therapeutically targeting autophagy is a promising approach to treat persistent inflammation and improve immune control of HIV replication.

## Graphical abstract

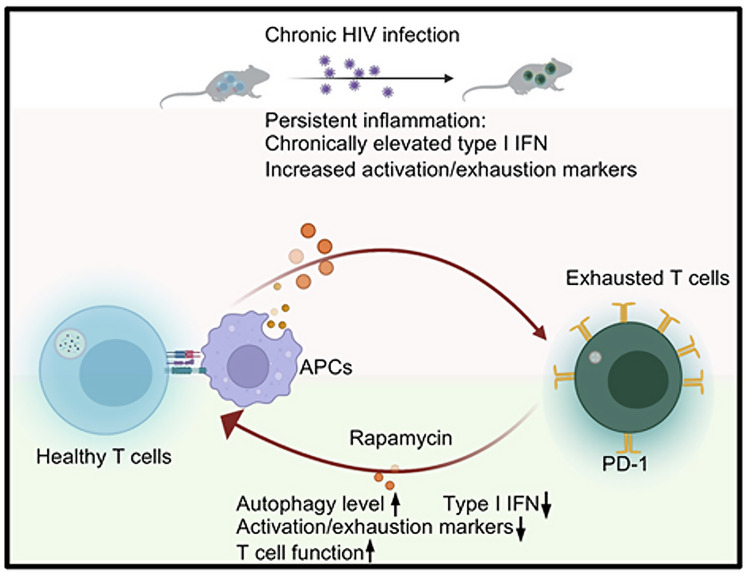

## Introduction

HIV-1–specific CD8^+^ cytotoxic T lymphocytes are essential in suppressing HIV replication and eliminating HIV-infected cells (1–3). However, during chronic HIV infection, persistent inflammation and viral escape drive T cell exhaustion, which is characterized by sustained high expression of inhibitory receptors (4–6), altered metabolism with inefficient bioenergy (7), changed transcriptional expression and epigenetic landscape, and loss of effector function (8–10). Importantly, these deficiencies persist despite prolonged antiretroviral therapy (ART) that successfully suppresses viremia (3, 11, 12).

Although type 1 IFNs (IFN-Is) are critical for viral control during acute infection (13, 14), mounting evidence indicates that chronically elevated IFN-I signaling can lead to T cell dysfunction and exhaustion by inducing the expression of negative regulators (15–17) as well as attrition of activated T cells (18) during chronic infections and in the tumor microenvironment. Previously, we and others (19, 20) have demonstrated that in HIV-infected humanized mice, IFN-receptor IFNAR1 blockade after established chronic infection functionally rescued HIV-specific T cells, decreased hyperimmune activation, and reduced the size of HIV viral reservoirs in combination with ART. These results highlight the importance of targeting persistent inflammation for restoring antiviral immune responses. However, because IFN-Is are key regulators in both antiviral innate and adaptive responses, blocking IFN receptors to IFN-Is may also be deleterious for viral immune control (21, 22). Thus, other approaches to safely curb persistent activation are needed.

Macro-autophagy (herein referred to as autophagy) is a homeostatic mechanism involved in the disposal of damaged organelles, such as mitochondria (23, 24), as well as eliminating intracellular pathogens (25, 26). It is a conserved cellular process critical for maintaining cellular integrity and metabolism (27). Degradation of proteins by autophagy is critical to maintaining cell function during cellular stress, such as nutrient deprivation caused by pathogen replication (28, 29). In addition to maintaining cellular integrity, autophagy has also been linked to both innate and adaptive immunity (30–34). Innate immune responses can activate autophagy (35), whereas autophagy regulates innate immune responses by modulating the secretion of immune mediators as well as removing endogenous inflammasome agonists (30, 33, 36). Autophagy also plays critical roles in adaptive immunity by modulating antigen processing and presentation (31, 37), lymphocyte development, homeostasis, activation, and survival (33, 38–40).

HIV-1 alters various stages of autophagy in both infected and bystander cells. For example, HIV-1 Nef protein interacts with Beclin 1 and impairs the maturation of autophagy in infected macrophages (41). HIV-1 Tat inhibits autophagy in bystander macrophages (42) and neurons (43). HIV-1 inhibition of autophagy in DCs impairs innate and adaptive immune responses and enhances cell-associated viral transmission to CD4^+^ T cells (44, 45). Interestingly, peripheral blood from HIV controllers demonstrated a significantly greater amount of autophagic vesicles and greater expression of autophagic markers as compared with typical progressors, suggesting that a greater level of autophagy activity can be beneficial for controlling HIV-1 replication (46). However, how autophagy affects IFN-I signaling and antiviral T cell responses in the pathogenesis of HIV-1 infection is largely unknown. In this study, we investigated the relationship between autophagy and IFN-I signaling in the context of HIV-1 infection and evaluated therapeutic potentials of autophagy inducers to reduce persistent inflammation and improve T cell function during HIV infection.

## Results

### Inhibition of autophagy by ATG5 disruption or bafilomycin A1 leads to increased IFN-I signaling in THP-1 cells.

**Figure 1:**
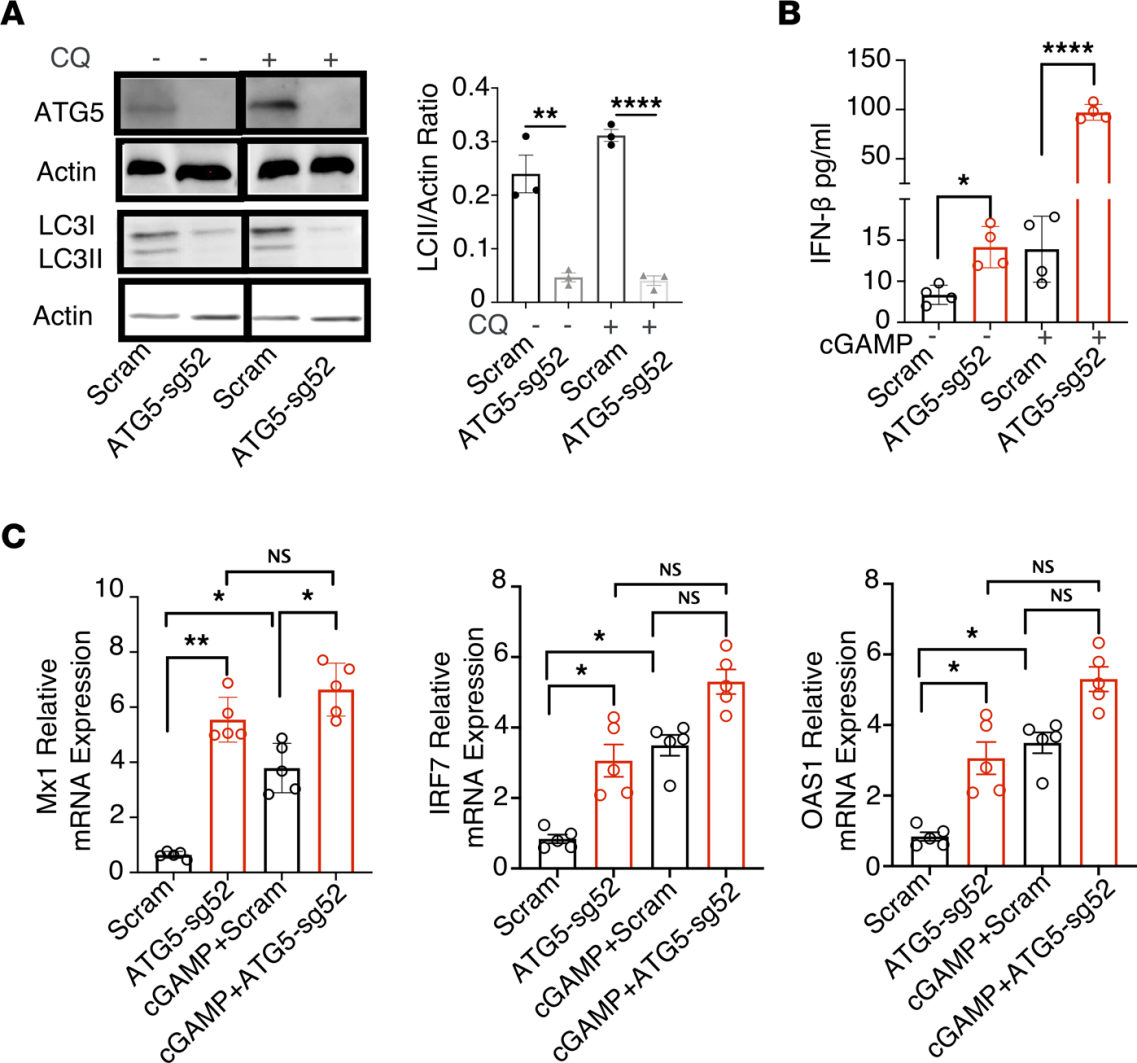
CRISPR/CAS9–mediated ATG5 disruption led to increased IFN-I signaling in THP cells. THP-1 cells were transduced with lentiviral particles containing sgRNA52 targeting ATG5 or scrambled sgRNA and subsequently incubated with puromycin to isolate stable cell lines. (**A**) THP-1-sg52 and scrambled control cell lines were incubated for 6 hours in the absence or presence of 10 μM chloroquine (CQ) to block lysosomal degradation. Whole-cell lysates were collected and ATG5 and LC3 expression was analyzed by Western blotting. β-Actin expression was assessed as a protein-loading control for ATG5 (top 2 blots from same gel) and LCI-II (bottom 2 blots from same gel). The LC3-II/actin ratio was analyzed by ImageJ. (**B**) IFN-β expression was measured in cell-culture supernatant of scrambled control or THP-1 ATG5-sg52 cells with or without cGAMP stimulation. (**C**) Relative RNA expression level of *MX1*, *IRF7*, *and OAS* in scrambled or THP-1 ATG5-sg52 cells with or without cGAMP stimulation. Data show the mean values of 3–5 independent experiments ± SEM (represented by error bars) *P < 0.05; **P < 0.01, ****P < 0.0001, Kruskal-Wallis with Dunn’s test.

Monocytes, macrophages, and DCs are major IFN-I–producing cells in the course of viral infection and play essential roles in driving immunopathogenesis during chronic viral infection (20, 47). Thus, using the monocytic cell line THP-1, we first investigated how autophagy regulates IFN-I responses. We generated autophagy-impaired THP-1 cells by using CRISPR/Cas9 to disrupt expression of autophagy-related gene 5 (ATG5), an indispensable component of autophagic vesicle formation. As shown in Figure 1A, ATG5 disruption led to reduced protein expression of ATG5 in the presence or absence of autophagy and the lysosomal inhibitor chloroquine in ATG5-sg52 cell lines. Importantly, we observed decreased microtubule-associated protein 1A/1B-light chain 3 (LC3)–II expression and a lower ratio of LC3-II/actin level in ATG5-sg52 cells by immunoblotting (Figure 1A) with or without chloroquine treatment, indicating impaired autophagosome formation in ATG5-sg52 cells (48, 49). Intriguingly, we observed increased production of IFN-β1 in ATG5-sg52 cells without stimulation, and this was further elevated with stimulation by 2′3′–cyclic GMP-AMP (c-GAMP), which triggers stimulator of IFN genes (STING)–dependent IFN-I responses by binding to DNA-sensor cGAMP synthase (Figure 1B) (50, 51).

To further investigate whether impaired autophagy affects expression of IFN-I–stimulating genes (ISGs), we measured the RNA expression of ISGs MX1, IRF7, and OAS1 and internal control HPRT1 by real-time PCR in scrambled control and THP-1 ATG5-sg52 cells in the presence and absence of 2′3′-cGAMP activation. As shown in Figure 1C, compared with the scrambled control, THP-1 ATG5-sg52 cells have spontaneous, elevated ISG expression in the absence of stimulation, which is further increased upon stimulation.

Bafilomycin A1 (BafA1) is a known inhibitor of the later stage of autophagy, inhibiting fusion between autophagosomes and lysosomes (52). To examine if inhibition of late-stage autophagy also affects IFN-I signaling, THP-1 cells were treated with BafA1 (50 nM) for 2 days, and we measured expression levels of ISGs MX1, IRF7, OAS1, and internal control HPRT1 by real-time PCR. As shown in Supplemental Figure 1 (supplemental material available online with this article; https://doi.org/10.1172/jci.insight.159136DS1), without additional stimulation, BafA1 treatment alone led to significant elevation of ISGs as compared with the control. Overall, these results indicate that impairment of autophagy by either ATG5 gene disruption or inhibitor BafA1 could lead to elevated IFN-I signaling, suggesting a crucial role of autophagy in regulating IFN-I production.

### Induction of autophagy by rapamycin or spermidine decreases IFN-I responses in monocytes and macrophages and is dependent on ATG5.

**Figure 2:**
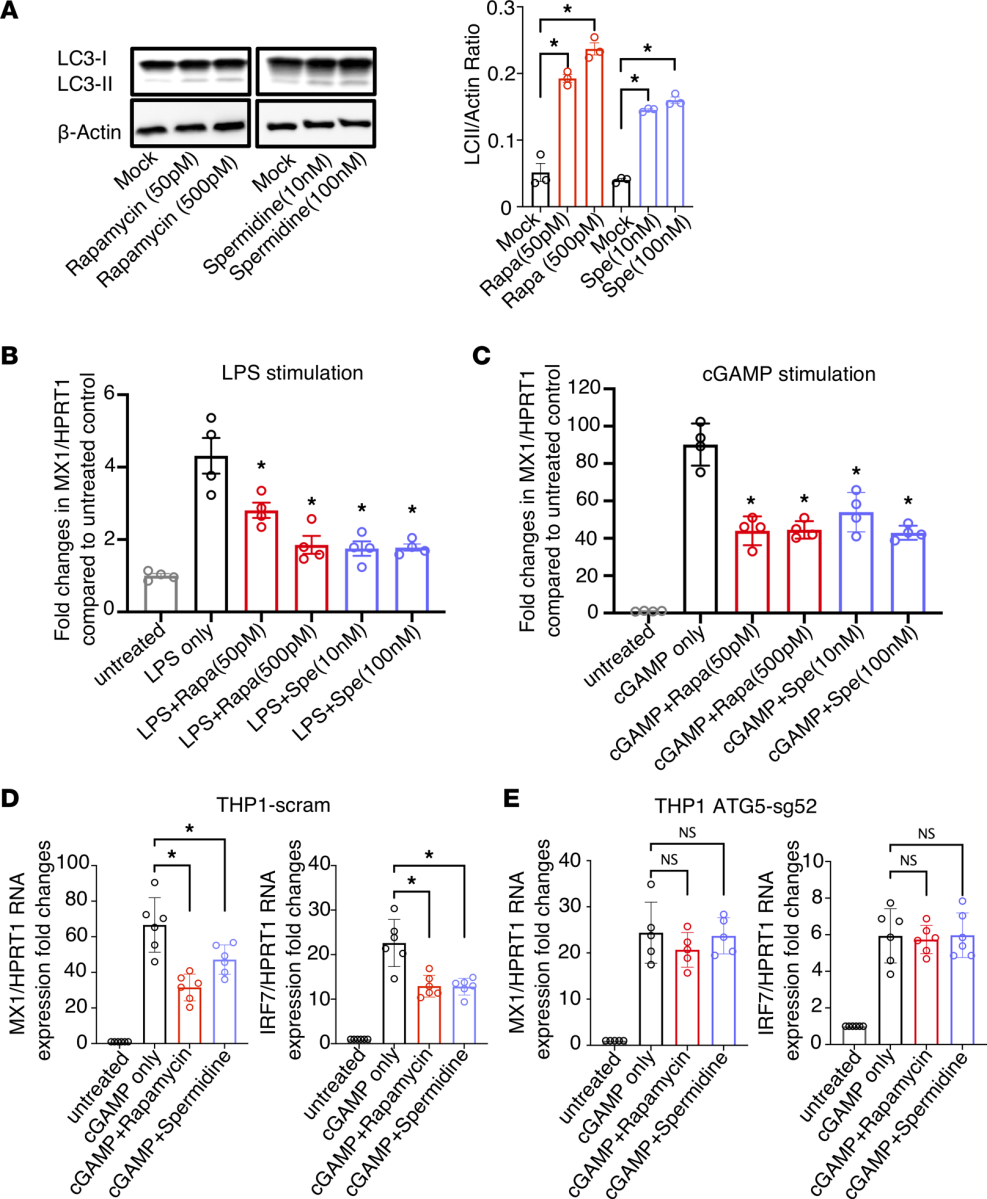
Autophagy induction by rapamycin (Rapa) reduces IFN-I signaling in activated macrophages and THP1 cells and is dependent on ATG5. CD14^+^ monocytes were sorted from healthy primary PBMCs with CD14 microbeads and differentiated into macrophages with macrophage colony-stimulating factor at 10 ng/mL for 3 days. Afterward, cells were treated with the autophagy inducers rapamycin (50 pM and 500 pM) or spermidine (Spe; 10 nM and 100 nM) for 2 days. (**A**) Autophagy flux was measured by western blotting for LC3 and actin. The ratio of LC3-II/actin was calculated by ImageJ. After rapamycin or spermidine treatment, cells were stimulated by (**B**) LPS or (**C**) cGAMP for 6 hours; the ISG MX1 and internal control HPRT1 were measured by real-time PCR. (**D** and **E**) THP-1 scram (**D**) or THP-1 ATG5-sg52 (**E**) cells were treated with 50 pM rapamycin or 100 nM spermidine for 2 days and followed by cGAMP stimulation for 6 hours. The ISGs MX1 and IRF7 and the internal control HPRT1 were measured by real-time PCR. Data are reported as the mean values of 3–5 independent experiments ± SEM. *P < 0.05, Kruskal-Wallis analysis with Dunn’s test.

Next, we examined if induction of autophagy can reduce IFN-I signaling using 2 autophagy inducers, rapamycin and spermidine, that target distinct components of the autophagy pathway. Rapamycin, an inhibitor of mTOR, is a potent inducer of autophagy and has been tested in diverse cells and animal models for autophagy induction (53). Spermidine, in contrast, activates autophagy via mTOR-independent pathways (54–56). To examine if autophagy induction affects IFN-I signaling in primary macrophages, we treated primary monocyte-derived macrophages with rapamycin or spermidine for 2 days, followed by stimulation with either LPS or 2′3′-cGAMP for 6 hours. Prior to stimulation, we confirmed increased autophagy flux by rapamycin or spermidine in macrophages as measured by LC3-II/actin ratio (Figure 2A). After stimulation, we observed that elevation of ISG MX1 level was significantly reduced by rapamycin or spermidine treatment for both LPS-stimulated (Figure 2B) and cGAMP-stimulated (Figure 2C) macrophages, suggesting that autophagy induction via distinct pathways can effectively lower LPS or 2′3-cGAMP–stimulated ISG expression in primary macrophages.

To further investigate whether rapamycin or spermidine suppression of IFN-I signaling is dependent on autophagy induction, we treated either scrambled control or autophagy-deficient ATG5-sg52 THP1 cells with either mock, 2′3′-cGAMP only, 2′3′-cGAMP with rapamycin, or 2′3′-cGAMP with spermidine. Similar to what we observed in primary macrophages, rapamycin and spermidine reduced activation of ISG MX1 by 2′3′-cGAMP stimulation in control THP1 cells (Figure 2D). However, rapamycin and spermidine suppression of 2′3′-cGAMP activation was blunted in ATG5-sg52 cells (Figure 2E), further demonstrating that rapamycin and spermidine downregulation of IFN-I is dependent on ATG5.

### Combining rapamycin with ART decreases chronic IFN-I–mediated inflammation and reduces viral RNA in HIV-1–infected humanized mice.

Anti-HIV T cell functions are critical for controlling HIV replication. However, during chronic infection, HIV immune invasion and persistent inflammation lead to dysfunctional HIV-specific T cells that are defective in eliminating HIV-infected cells (57). Consequently, T cell exhaustion remains one of the major barriers for achieving sustained immune surveillance for HIV infection (58). This leads to the idea that alleviating chronic activation could halt disease progression and restore immunological defects. We (20) and others (19) showed that persistent IFN-I signaling is one of the culprits that drives T cell exhaustion and blocking IFN-I receptor could restore anti-HIV T cell responses and lower the viral load or reservoir in vivo. However, because IFN-Is are key regulators in both adaptive and innate responses, safer approaches to curb persistent activation are needed.

**Figure 3:**
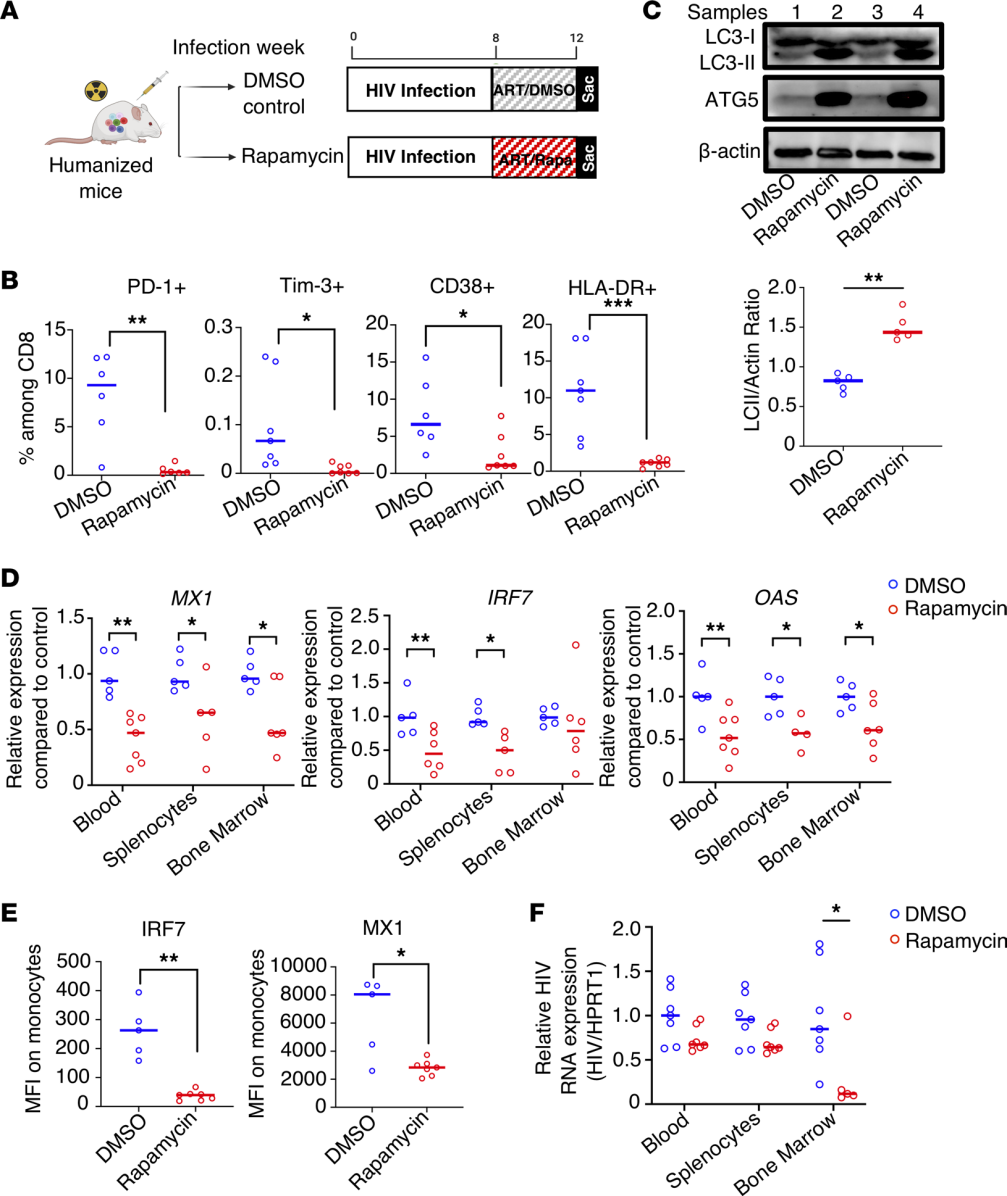
Combination of ART and autophagy inducer rapamycin (Rapa) treatment effectively decreases inflammation, ISG expression, and viral replication in HIV-infected humanized BLT mice. (**A**) Eight weeks after immune reconstitution, BLT humanized mice were infected with HIV_NFNSXL9_ for 8 weeks. Afterward, mice were treated with ART and rapamycin or DMSO control for 4 weeks before necropsy. (**B**) PD-1, TIM-3, CD38, and HLA-DR expression was measured by flow cytometry (quantitatively by gating of percentages positive ± SEM) on peripheral blood CD8^+^ T cells from rapamycin or control mice (*n* = 6–7 per group). (**C**) Autophagy flux was detected by Western blotting of LC3-I, LC-II, ATG5, and actin using pooled splenocytes from DMSO or rapamycin-treated groups at necropsy (*n* = 5 per group). The ratio of LC3-II/actin was calculated by ImageJ. (**D**) Expression levels of human ISGs *MX1*, *OAS1*, and *IRF7* in multiple lymphoid tissues from humanized BLT mice after treatment were measured by real-time PCR (*n* = 6–7 per group). (**E**) Splenocytes from HIV-1–infected mice treated with ART and rapamycin or DMSO were isolated and stained with intracellular Abs against human IRF7 and MX1. MFIs of the ISGs IRF7 and MX1 on human monocytes were measured by flow cytometry (*n* = 5–7 per group). (**F**) Relative HIV cellular RNA/HPRT1 expression from multiple lymphoid tissues after the indicated treatment, as compared with control blood. (*n* = 5–7 per group). Each dot represents an individual mouse; horizontal bars indicate median values. *P < 0.05, **P < 0.01, ***P < 0.001, Mann-Whitney *U* test.

To observe if the autophagy inducer rapamycin can treat persistent inflammation during HIV infection in vivo, we examined the effect of rapamycin treatment in humanized mice infected with HIV-1. Rapamycin is an FDA-approved drug for the prevention of transplant rejection. It is a well-characterized autophagy inducer and has an excellent safety profile, including in patients with HIV (59, 60). Humanized bone marrow/liver/thymus (BLT) mice were constructed and infected with HIV-1 for 8 weeks. Afterward, mice were given ART with either rapamycin or DMSO control for 4 weeks, as outlined in Figure 3A. After HIV infection, and prior to rapamycin treatment and ART, we observed gradual elevation of exhaustion marker PD-1 (Supplemental Figure 2A, 0–8 weeks; supplemental material available online with this article; https://doi.org/10.1172/jci.insight.159136DS1) and ISG MX1 (Supplemental Figure 2B, 0–8 weeks) in the blood of infected animals as expected. We did not observe any differences in PD1, MX1 expression, or viral load (Supplemental Figure 2C, 0–8 weeks) between rapamycin or DMSO treatment groups prior to treatment initiation. In contrast, after ART with rapamycin or DMSO control, as shown in Figure 3B, we observed reduced expression levels of exhaustion markers PD-1 and Tim-3 and activation markers CD38 and HLA-DR in CD8^+^ T cells from peripheral blood in animals that received both rapamycin and ART. As shown in Figure 3C, we also observed increased ATG5 expression and LC3-II/actin expression in mice that received rapamycin treatment, suggesting increased autophagy flux by rapamycin in vivo.

To investigate the impact of rapamycin treatment on IFN-I signaling, ISGs were measured at the terminal time point by both real-time PCR (Figure 3D) and flow cytometry (Figure 3E) to measure the changes in RNA and protein expression level. Consistent with the previous in vitro data, ART combined with rapamycin led to significantly decreased expression of ISGs such as MX1, IRF7, and OAS1 in multiple lymphoid tissues (blood, spleen, and bone marrow) (Figure 3D). There was also reduction of ISG IRF7 and MX1 protein levels in human monocytes from spleen of mice receiving combined ART and rapamycin treatment (Figure 3E).

Plasma viremia for all mice was undetectable at the time of necropsy 4 weeks after ART initiation (Supplemental Figure 2C). However, we observed a trend of lower cellular viral RNA level in blood, splenocytes, and bone marrow in the ART and rapamycin-treated group (Figure 3F) as compared with ART and DMSO control group.

To investigate whether rapamycin leads to changes in the composition of immune cell types with different abilities to express ISGs (61, 62), we investigated alterations in the percentages of T cells (CD45^+^CD3^+^CD20^–^), B cells (CD45^+^CD20^+^CD3^–^), and monocytes (CD45^+^CD14^+^CD3^–^CD20^–^) among total human CD45^+^ lymphocytes in spleens of humanized mice after treatment. Results show that rapamycin treatment did not affect major lymphocyte composition in humanized mice (Supplemental Figure 3A). In addition, we performed T cell subset analysis to investigate whether rapamycin treatment has a differential impact on T cell subsets during infection (63, 64) or inhibition of T cell proliferation (65–67). We found rapamycin treatment did not significantly change CD4 or CD8 percentages in the peripheral blood in HIV-infected mice (Supplemental Figure 3B). Importantly, we observed downregulation of PD-1 expression in peripheral blood CD8 T cells across all non-naive subsets, including central memory (CD45RA^–^CCR7^+^), effector memory (CCR7^–^CD45RA^–^), and terminally differentiated effector memory T cells (CD45RA^+^CCR7^–^) T cell subsets, whereas the naive subset (CD45RA^+^CCR7^+^) had low PD-1 expression levels with or without rapamycin treatment (Supplemental Figure 3C). Last, we observed an increase of naive CD4 T cells in bone marrow of mice treated with rapamycin (Supplemental Figure 3D), which may have contributed to reduced viral RNA in bone marrow (Figure 3F). These observations led us to further evaluate the impact of rapamycin on viral load before ART and viral rebound after ART withdrawal.

### Rapamycin treatment alone reduces IFN-I inflammation and viral load; combined with ART, it improves antiviral T cell function and reduces viral rebound after ART discontinuation.

**Figure 4:**
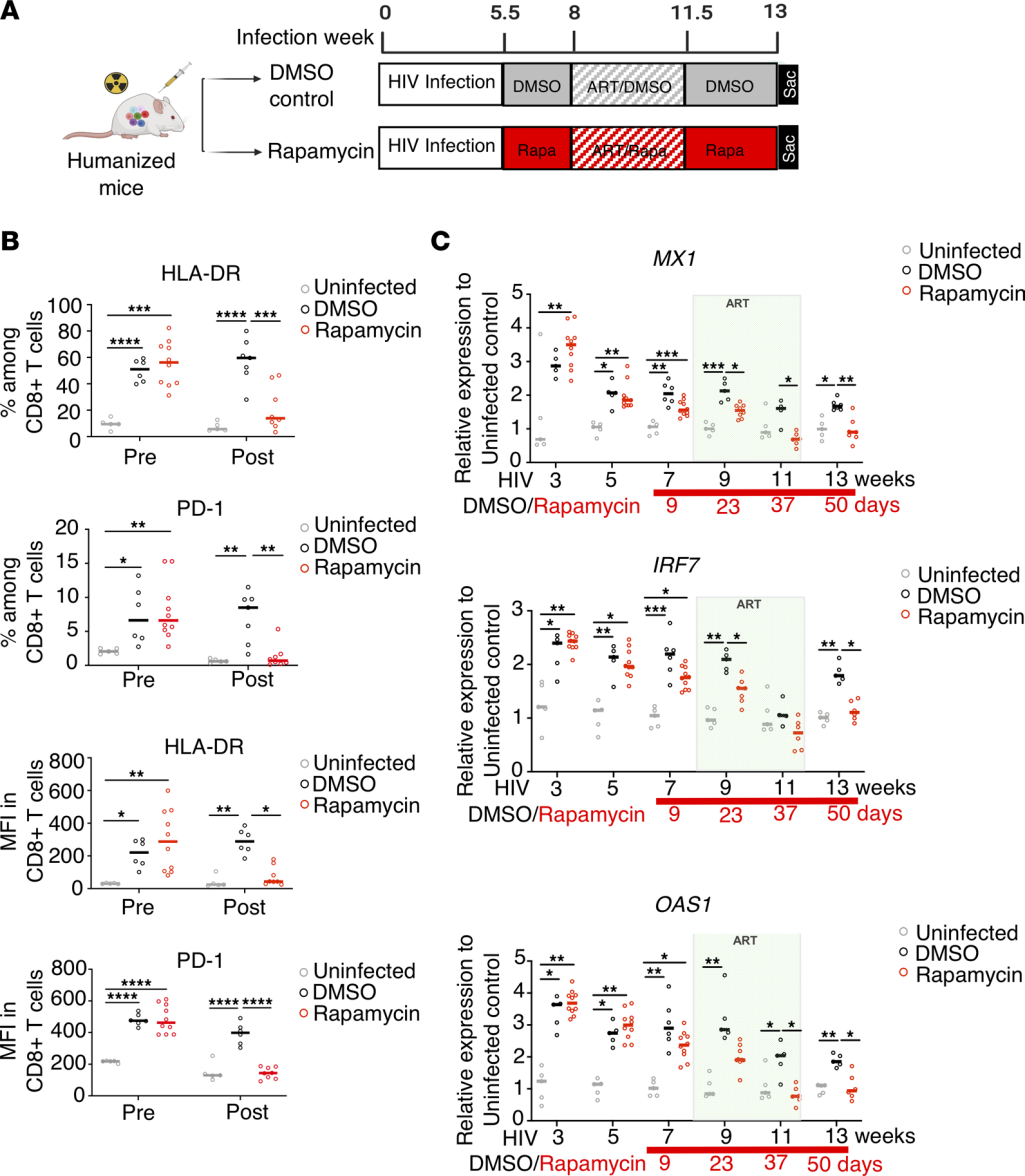
Autophagy inducer rapamycin (Rapa) effectively decreases inflammation and reduces IFN-I signaling. (**A**) At 5.5 weeks after HIV infection, BLT humanized mice were treated with rapamycin or DMSO control for 2 weeks. Afterward, while continuing rapamycin or DMSO treatment, mice were treated with ART for 3.5 weeks, followed by ART interruption for 10 days. (**B**) HLA-DR and PD-1 expression was measured by flow cytometry (quantitatively by gating of percentages positive) on peripheral blood CD8^+^ T cells before and after rapamycin or control treatment (*n* = 5–8 per group) prior to ART. (**C**) Expression levels of the ISGs MX1, OAS1, and IRF7 in human PBMCs from humanized BLT mice after treatment were measured by real-time PCR throughout HIV-1 infection and from rapamycin- or DMSO control–treated mice in comparison with uninfected animals (*n* = 5–8 per group). Each dot represents an individual mouse; horizontal bars indicate median values. *P < 0.05, **P < 0.01, ***P < 0.001, ****P < 0.0001, LMM.

To further investigate the effects of rapamycin treatment on inflammation and antiviral T cell function in the presence and absence of ART, we first treated chronically HIV-infected humanized BLT mice with rapamycin (or DMSO control) for 2 weeks prior to ART, followed by ART with continued rapamycin (or DMSO) for 3.5 weeks. After plasma viremia became undetectable, ART was interrupted to evaluate viral rebound (Figure 4A). Prior to ART, rapamycin treatment significantly reduced both activation-marker HLA-DR and exhaustion-marker PD-1 expression on CD8^+^ T cells (Figure 4B), suggesting that rapamycin treatment alone in HIV-1–infected humanized mice could lead to reductions in activation- and exhaustion-marker expression among CD8^+^ T cells.

Next, we investigated longitudinal expression level of ISGs MX1, IRF7, and OAS in HIV-1–infected mice treated with rapamycin (or DMSO control), followed by ART and discontinuation of ART. As shown in Figure 4C, HIV infection led to chronic elevation of IFN-I signaling, even in the presence of ART (Figure 4C, comparing the HIV-infected, DMSO-treated group versus uninfected controls). Rapamycin treatment alone significantly reduced IFN-I signaling (Figure 4C, week 7), which was further reduced when combined with ART to levels similar to those in uninfected controls (Figure 4C, weeks 9–11). After ART discontinuation and viral rebound, we observed significant elevation of ISGs in the DMSO control–treated mice as compared with uninfected control mice, whereas the ART and rapamycin combined-treatment group maintained low levels of ISGs (Figure 4C, week 13). Overall, ISG expression was significantly reduced in the rapamycin group as compared with the DMSO group (MX1: *P* < 0.0001; IRF7: *P* = 0.0167; OAS1: *P* = 0.0007, by linear mixed model [LMM]).

In addition, we investigated rapamycin’s effects on different immune cell types and T cell subsets. Rapamycin treatment did not alter the overall percentages of T cell, B cell, and monocytes subsets among total CD45^+^ lymphocytes (Supplemental Figure 4A). Rapamycin treatment also did not affect the percentages of CD4 and CD8 cells among T cells in HIV-infected mice (Supplemental Figure 4B). Last, apart from a slight increase of bone marrow–naive CD8 T cells, we did not observe a significant impact of rapamycin treatment on T cell subsets across different tissues (Supplemental Figure 4, C–E).

**Figure 5:**
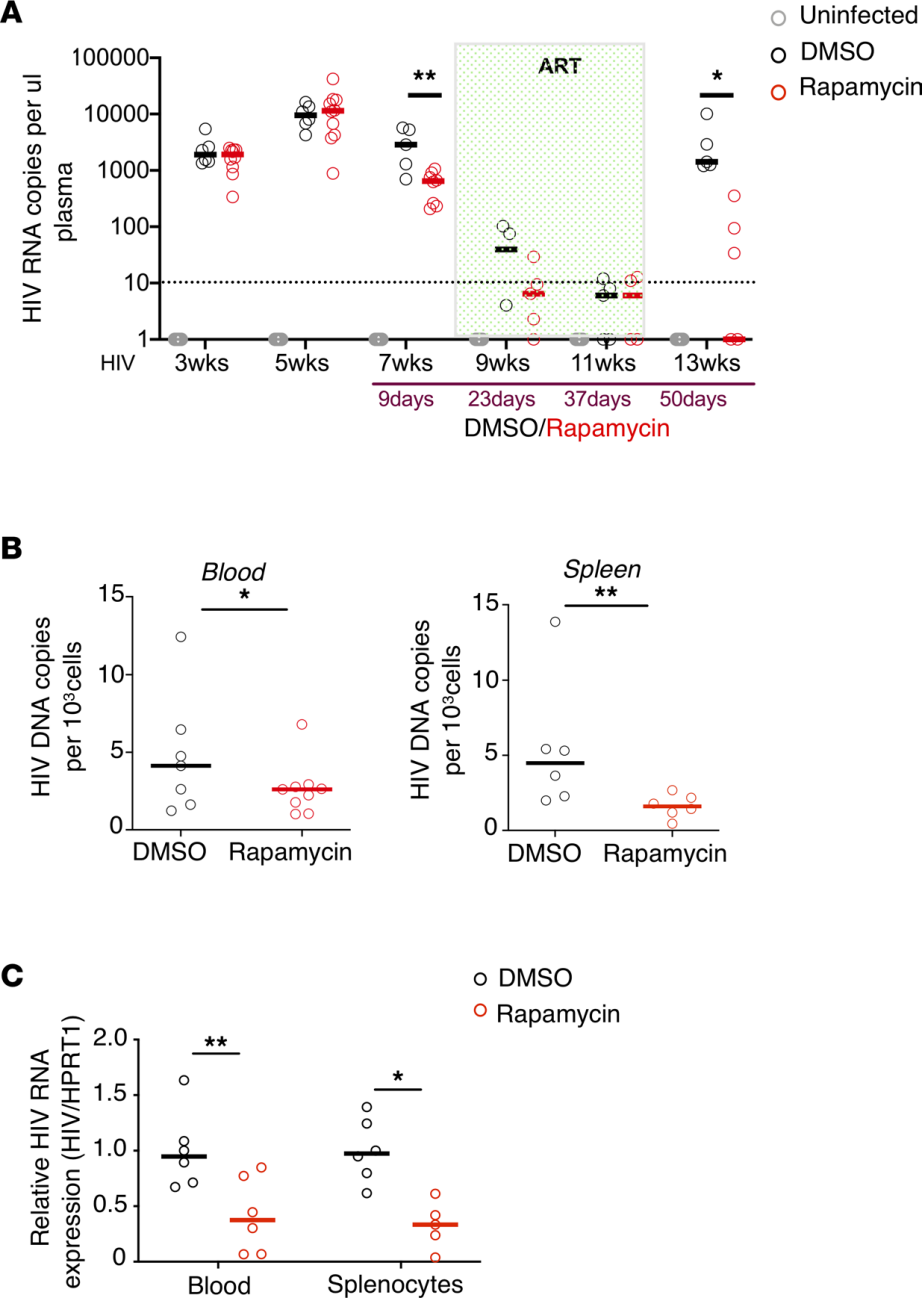
ART and autophagy inducer rapamycin cotreatment effectively reduces viral replication. (**A**) Longitudinal HIV viral load in plasma from humanized BLT mice after treatment were measured by real-time PCR. *P < 0.05, **P < 0.01, LMM. (**B**) HIV DNA copies per 10^3^ cells (measured by huRRP30 expression) from blood PBMCs or splenocytes as measured by real-time PCR (*n* = 6–8 per group). (**C**) Relative HIV cellular HIV/HPRT1 compared with control blood from at end point (*n* = 5–8 per group). Each dot represents an individual mouse; horizontal bars indicate median values. *P < 0.05, **P < 0.01, Mann-Whitney *U* test.

We next examined longitudinal changes in plasma viremia in treated mice. As shown in Figure 5A, prior to ART, 10 days of rapamycin treatment alone reduced plasma viremia as compared with the DMSO control (Figure 5A, week 7). Combination of rapamycin and ART led to faster viral suppression as compared with ART and DMSO control treatments (Figure 5A, weeks 9–11). Intriguingly, after ART withdrawal, the rapamycin-treated group had significantly lower plasma viremia after viral rebound (~1 log lower) (Figure 5A, week 13). Overall, we observed significantly lower viral load in the rapamycin group than in the DMSO group (*P* = 0.0002, by LMM). In addition, we also observed significantly lower levels of viral DNA (Figure 5B) and HIV RNA (Figure 5C) in both blood and spleen at necropsy after ART withdrawal, suggesting a reduction in overall viral replication in the rapamycin-treated group.

**Figure 6:**
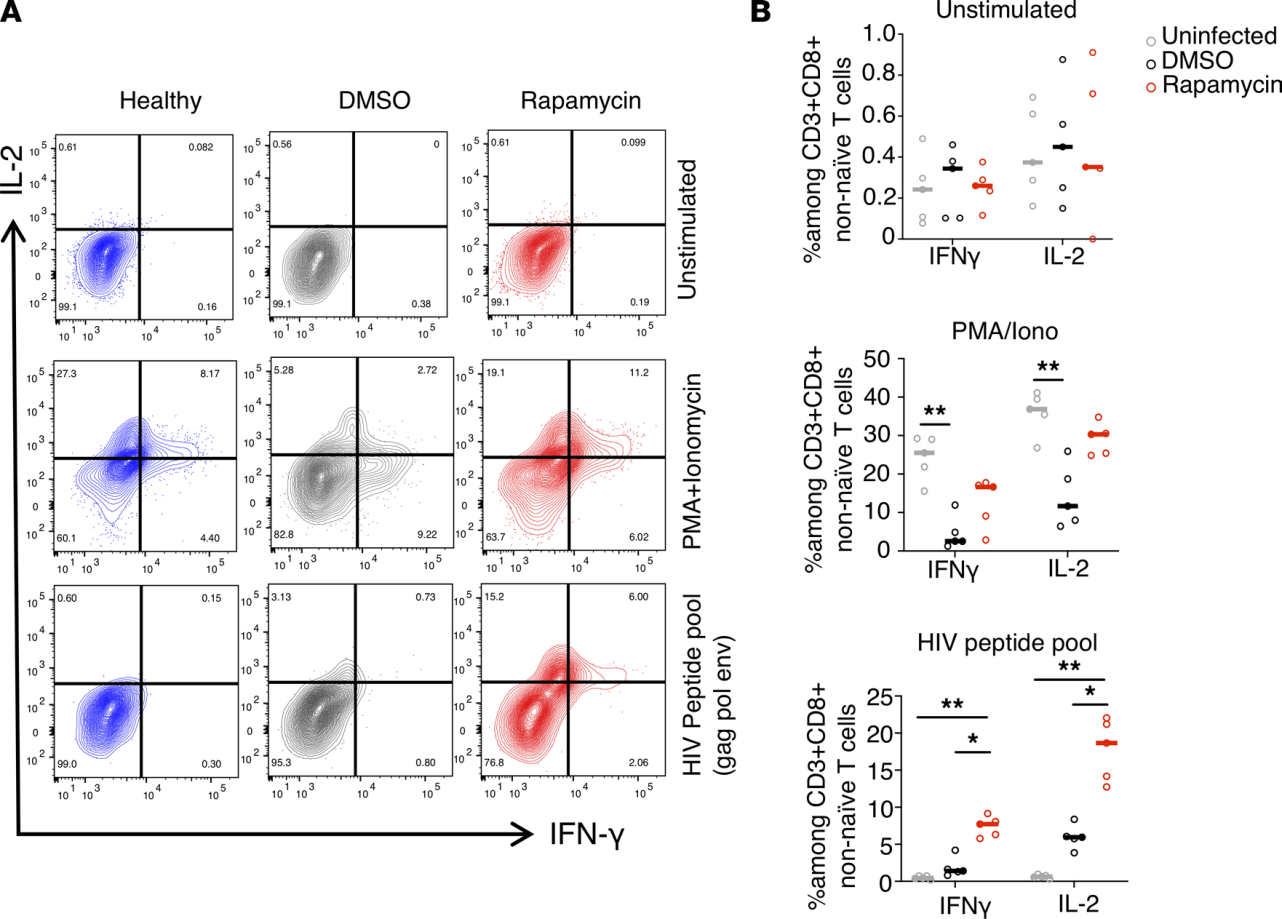
ART and autophagy inducer rapamycin cotreatment improve anti-HIV immune responses. Splenocytes from HIV-1–infected, DMSO-treated, or rapamycin-treated mice were stimulated with PMA or ionomycin or an HIV-1 clade B peptide pool (Pol, Gag, Env, and Nef), and production of IFN-γ by CD8 cells was measured by flow cytometry (representative of *n* = 4–6 per group). (**A** and **B**) Representative flow (**A**) and cytokine assay summary (**B**) showing percentage of IFN-γ^+^ and IL-2^+^ among CD3^+^CD8^+^ non-naive T cells from HIV-1–infected, DMSO-treated, or rapamycin-treated mice. (*n* = 4–6 per group). Each dot represents an individual mouse; horizontal bars indicate median values. *P < 0.05, **P < 0.01 Kruskal-Wallis analysis with Dunn’s test.

To investigate whether HIV-specific CD8^+^ T cell responses were improved in the ART and rapamycin combined-treatment group, we stimulated splenocytes from uninfected, rapamycin-treated, or DMSO control mice with either the mitogens PMA or ionomycin or an HIV clade B peptide pool (Gag, Env, Nef, and Pol). To control for potential enrichment effects of rapamycin on naive T cells (63, 64, 68), we closely examined non-naive CD8^+^ T cells, as shown in Supplemental Figure 5 (representative gating plots), Figure 6A (representative flow), and Figure 6B (flow summary). Compared with the uninfected control, non-naive CD8^+^ T cells from DMSO-treated infected mice produced significantly lower levels of pro-inflammatory IFN-γ and IL-2 cytokines after PMA or ionomycin stimulation, suggesting functional exhaustion of T cells. In contrast, non-naive CD8^+^ T cells from infected mice treated with rapamycin produced increased levels of pro-inflammatory cytokines in response to both PMA or ionomycin and HIV-1–specific peptide pool stimulation. As a whole, these data suggest that combination of rapamycin and ART improved HIV-specific and mitogen stimulation–induced T cell responses and is correlated with increased control of viral replication after ART withdrawal.

## Discussion

IFN-Is and autophagy are biological stress responses that are evolutionarily conserved (69, 70). Recent discoveries suggest that the crosstalk between autophagy and IFN-I signaling can be critical in regulating innate antiviral immunity (71). IFN-I and ISGs can regulate autophagy, whereas autophagy serves fundamental functions in eliminating excessive ISGs (72–74). However, how autophagy activities modulate IFN-I responses has yet to be fully elucidated. Our study provides strong evidence that autophagy can directly regulate IFN-I responses in monocytes and macrophages. We demonstrated that inhibition of autophagy by ATG5 disruption or BafA1 treatment in monocytic cell lines leads to spontaneous elevation of IFN-I signaling and further potentiates ISGs production in response to 2′3′-cGAMP stimulation. We found that induction of autophagy decreases by either rapamycin or spermidine treatment, both of which activate autophagy via distinct pathways (53, 54, 56, 75–77), downregulated ISG expression in stimulated primary macrophages. Importantly, we found the downregulation of ISGs by rapamycin and spermidine in THP1 is dependent on ATG5, suggesting that the effect of rapamycin or spermidine on ISG expression in these cell types is mediated by the process of autophagy. Our findings are consistent with those of recent studies showing that autophagy induction is involved in the IFN-I–signaling regulation mediated by cGAS–STING. Activation of STING by 2′3′-cGAMP induces autophagy, which, in turn, degrades phosphorylated STING, thereby tuning down IFN-I responses (78, 79). In addition, autophagy proteins can interact with cGAS and suppress IFN-I production via a STING-dependent pathway (80, 81). In combination, these findings strongly support direct regulation of IFN-I responses by autophagy.

To further evaluate the therapeutic potential of rapamycin to treat HIV infection, we investigated the effects of rapamycin in HIV-infected humanized mice in vivo. Rapamycin, an mTOR1 inhibitor, is a well-characterized, potent inducer of autophagy and was selected for our in vivo studies. Interestingly, rapamycin reduces inflammation, prolongs lifespan, and delays age-related diseases in diverse species (82). Treatment with rapamycin also reduces HIV persistence in recipients of a kidney transplant who also are infected with HIV-1 (60, 83). A recent study also demonstrated that everolimus and rapamycin limit intestinal HIV-1 transmission and reduce ongoing HIV-1 replication in intestinal CD4^+^ T cells in an autophagy-dependent manner (84). Therefore, rapamycin has been proposed as an immunologic strategy for HIV-1 remission and eradication beyond organ-transplant recipients (85). When we treated HIV-infected humanized mice with ART alone or combined ART and rapamycin treatments, we found that the autophagy level can be successfully induced by rapamycin treatment in vivo. Importantly, combined rapamycin and ART led to significant reduction in expression of T cell immune activation markers and ISGs. Although all mice had undetectable viremia at the time of necropsy, rapamycin and ART cotreated mice had a trend of lower HIV RNA levels in blood and lymphoid tissues compared with control mice.

We further examined the effects of rapamycin on HIV-1 replication before and after ART discontinuation. We found that prior to ART, rapamycin treatment alone significantly reduced expression of activation and exhaustion markers on CD8^+^ T cells. Combined with ART, rapamycin treatment accelerated reduction of viral load and further reduced residual elevated ISG expression that persisted in ART-treated mice to levels similar to those of healthy controls. Importantly, after ART withdrawal, rapamycin-treated mice had significantly reduced (1 log lower) viral rebound level as compared with DMSO control mice, with significantly lowered viral DNA and RNA levels in blood and splenocytes. Our study findings are consistent with those of a previous report showing that organ-transplant recipients with HIV infection who were treated with the rapamycin analog everolimus had significantly lower levels of viral RNA and immune activation markers (60). Importantly, previous studies in murine models of lymphocytic choriomeningitis virus infection (64) and a nonhuman primate study of vaccination (86) suggested that rapamycin treatment enhanced not only the quantity but also the quality of antigen-specific CD8 T cell responses, leading to enhanced memory formation of CD8 immunity (87). Consistent with those data, our ex vivo analysis showed that rapamycin treatment significantly improved cytokine production of non-naive CD8^+^ T cells in response to viral peptide stimulation, suggesting improved T cell functions could contribute to the reduced viral rebound. Taken together, our study demonstrates that in vivo treatment with rapamycin could lead to a reduction of IFN-I–mediated inflammation and improved T cell function, resulting in lower viral rebound and decreased HIV DNA and RNA levels in blood and tissues, potentially through its upregulation of autophagy.

Rapamycin is an inhibitor of mTOR, which is a nutrient-sensing master regulator for growth and cellular metabolism (88). Besides induction of autophagy, rapamycin also affects other signaling pathways, including glycolysis and cell growth (89). Rapamycin has concentration-dependent effects (87, 90–93). Considering the potential immunosuppressive effects of rapamycin at higher doses (94), we intentionally chose a low-dose rapamycin treatment regimen that has been shown to improve T cell responses (64) and extend the lifespan in murine models (95–97). We observed increased autophagy activity of PBMCs and improved anti-HIV T cell responses after rapamycin treatment in vivo. However, our study demonstrating autophagy-dependent reduction of IFN-I by rapamycin is limited to in vitro studies using THP-1 cell lines. Therefore, additional studies are needed to delineate the exact roles of autophagy in improving functions of exhausted T cells in vivo. In addition, the present study focuses on the application of autophagy inducers to reduce IFN-I–mediated inflammation rather than HIV-mediated impairment of autophagy. However, several studies have shown that HIV-1 modulates autophagy to facilitate efficient viral replication (45), and multiple HIV-1 proteins are reported to impair autophagy, including Tat (42), Nef (41, 98), Vpr (99), Vif (100). This connection is circumstantial, and additional studies are needed to understand how HIV infection affects autophagy in different immune cell types in vivo.

Nonetheless, our study revealed potentially novel and potential therapeutic effects of autophagy inducers in treating IFN-I–mediated chronic inflammation and improving antiviral T cell responses during HIV infection. Importantly, we did not observe enhancement of viral latency by rapamycin treatment (as evidenced in similar reduction in both HIV DNA and RNA in rapamycin-treated mice). Interestingly, rapamycin can be used to reduce cytokine-associated toxicity by latency-reversal reagents without affecting reactivation of latent HIV (101). We also did not observe an impact of low-dose rapamycin on HIV expression on stimulated primary CD4 T cells that were infected with HIV in vitro (Supplemental Figure 6). This suggests that rapamycin can be used in combination with other immunologic or latency reversal–based HIV cure strategies. Therefore, additional studies should be carried out to further explore the therapeutic effects of a variety of autophagy inducers on treating HIV-1 infection and pathogenesis (102). Our in vitro and in vivo studies provide for future clinical trials critical insights into the mechanism of action of rapamycin in suppressing HIV infection and reducing HIV-mediated chronic inflammation.

In conclusion, our study revealed important regulatory cross talk between autophagy and IFN-I immune responses and demonstrated the therapeutic potentials of autophagy inducers for treating HIV infection and comorbidities that are caused by chronic immune activation. Last, we envision that the IFN-I modulatory effects of autophagy inducers reported in this study and others could be used beyond treating HIV infection to treat other chronic diseases involving persistent inflammation, such as severe and long-term COVID-19 (103–105) and age-associated diseases (106, 107).

## Methods

### Lentiviral production.

The CRISPR/Cas9 All-in-One scrambled control or ATG5 lentiviral vectors (Abmgood) and the lentivirus-based GFP-CAR vectors were produced in 293FT cells using the Lipofectamine 2000 reagent (Invitrogen). Briefly, 293FT cells were co-transfected simultaneously with CRISPR/Cas9 All-in-One ATG5 lentiviral vector or CAR vectors with pCMV.ΔR8.2.Δvpr packaging construct and the pCMV-VSV-G envelope protein plasmid, as previously described (20, 108). Supernatant was collected from transfected 293FT cells 48 hours after transfection, filtered using a 0.45 μm sterile filter, and concentrated by ultracentrifugation using a Beckman SW32 rotor at 154,000*g* at 4°C. Medium was aspirated, and the pellet was resuspended with PBS and stored at –80°C.

### Generation of ATG5-knockdown THP-1 cells.

To generate ATG5 knockdown cells, THP-1 cells were infected with CRISPR/Cas9 All-in-One lentiviral vector at an MOI of 2 in a medium supplemented with polybrene (4 μg/mL) overnight. Two consecutive rounds of infections were performed to improve efficiency. Cells were treated with 2.5 mg/mL puromycin 48 hours after transduction. Polyclonal stable cell-line libraries were established after 2 weeks of drug selection. The target sequence was sg-52:TGATATAGCGTGAAACAAGT. After selection, the ATG5-knockdown THP-1 cell line was cultured in RPMI medium containing 10% FBS and puromycin at a final concentration of 1 mg/mL.

### Cytokine assay.

We assessed purified splenocytes from BLT mice for production of IFN-γ, or IL-2 by intracellular staining and flow cytometry. In brief, for nonspecific stimulation, splenocytes from BLT mice were stimulated ex vivo with PMA (50 ng/mL) and ionomycin (1 μM; Sigma-Aldrich) for 6 hours in the presence of GolgiPlug and Golgi Stop (BD Biosciences). For antigen-specific stimulation, splenocytes from humanized mice were stimulated ex vivo with HIV-1 clade B Env, Gag, Pol peptide pool (NIH AIDS Reagent Program, www.aidsreagent.org) overnight and an additional 6 hours of GolgiPlug and Golgi Stop treatment. Cells were then fixed and permeabilized with Cytofix/Cytoperm buffer (BD Biosciences), and intracellular staining was then performed.

### Real-time PCR.

To measure HIV plasma viremia, viral RNA was extracted from plasma and 1-step real-time PCR was performed using the TaqMan RNA-to-Ct 1-Step Kit (Thermo Fisher Scientific) with the following primers and probe, as previously described (20): HIV-1 forward primer: 5′-CAATGGCAGCAATTTCACCA-3′; HIV-1 reverse primer: 5′-GAATGCCAAATTCCTGCTTGA-3′; HIV-1 probe: 5′-[6-FAM]CCCACCAACAGGCGGCCTTAACTG [Tamra-Q]-3′; and single-tube TaqMan Gene Expression Assays (Thermo Fisher Scientific): human HPRT1 (Hs01003267_m1), MX1 (Hs00895608_m1), IRF7(Hs01014809_g1), OAS1 (Hs00973635_m1). To measure the levels of cell-associated HIV RNA and the ISGs MX1 and OAS1, with HPRT1 as an internal control, splenocytes and PBMCs were harvested for RNA extraction and the making of cDNA, using the High-Capacity cDNA Reverse Transcription Kit (Thermo Fisher Scientific).

### IFN-I quantification.

IFN-I levels in cell-culture supernatant samples were measured using the LEGENDplex Human Type 1/2/3 Interferon Panel 5-plex Panel kit (BioLegend), measuring human proteins, including IFN-α, IFN-β, IFN-λ1, IFN-λ2/3, and IFN-γ. Samples were acquired on a BD FACSFortessa II and analyzed using LEGENDplex software (BioLegend).

### Western blotting.

Approximately 2 million cell samples were lysed in 0.5 mL of ice-cold T-PER tissue protein extraction buffer (Thermo Scientific) with protease and phosphatase inhibitors (Sigma). Total protein (30 μg) was separated by SDS-PAGE using Criterion XT Precast Gel (Bio-Rad) and blotted with Abs. Abs ATG5, LC3-B, and actin were obtained from Abcam, and Abs pSTING and STING were purchased from Cell signaling. Western blots were quantified using ImageJ software (NIH).

### Humanized BLT mice.

Humanized mice were constructed as described previously (15, 30). In short, CD34^+^ cells were purified via magnetic cell sorting with CD34 microbeads (Miltenyi Biotec) from freshly obtained fetal liver tissues. NOD–SCID–common γ-chain KO (cγ^–/–^) mice were sublethally irradiated (2.7 Gy) before the surgery and implanted with fetal liver and thymus derived from the same donor as the CD34^+^ cells. Afterward, mice were injected with 0.5 million to 1 million CD34^+^ cells. After 8 to 10 weeks, each mouse was bled retro-orbitally to check human immune-cell engraftment. Each donor tissue can be used to construct 15 to 25 mice. Mice that had more than 50% human lymphocytes in the PBMCs were used for HIV infection and further experiments. A standard, healthy, uninfected humanized BLT mouse contains 0.5 million to 2 million human lymphocytes per milliliter in the blood and 10 million to 30 million human lymphocytes per milliliter in the spleen (109).

### Rapamycin treatment and ART.

For rapamycin treatment, mice were injected i.p. with 0.5 mg/kg rapamycin (Sigma) 3 times a week. An oral ART regimen was used by mixing 3 antiretroviral drugs directly into food: emtricitabine, tenofovir, and raltegravir. All drugs were provided by Gilead. Average daily drug doses were as follows: emtricitabine, 87.5 mg/kg/day; tenofovir, 131 mg/kg/day; and raltegravir, 175 mg/kg/day each (calculated by average daily food intake of 3.5 g and average weight of 20 g per mouse).

### Abs and flow cytometry.

The following Abs were used in flow cytometry: CD45 (clone HI30), CD3 (clone OKT3), CD4 (clone RPA-T4), CD8 (clone SK1), CD38 (clone HIT2), HLA-DR (clone L240), CD14(clone RMO52), CD45RA (clone HI100), CD62L (clone DREG-56), IFN-γ (clone 4S.B3), IL-2 (clone MQ1-17H12), IFIT3 (clone OTI1G1), IFI6 (clone 1G7), IRF7 (clone 12G9A36), MX1(clone EPR19967), PD-1 (clone ebioJ105), TIM-3 (clone F38-2E2), and pSTING (clone E9A9K). LIVE/DEAD Fixable Yellow Dead Cell Stain Kit (Invitrogen) was used. Abs for cell surface markers and intracellular markers were conjugated to FITC, phycoerythrin (PE), PerCP-Cy5.5, PE-Cy5, PE-Cy7, electron coupled dye (ECD), allophycocyanine (APC), APC–eFluor780, Alexa Fluor 700, eFluor450, Pacific Orange, or Pacific Blue in the appropriate combination. The cells were acquired using an LSRFortessa flow cytometer and FACSDiva software (BD Biosciences). Data were analyzed using FlowJo software. At least 1000 cells were acquired for each analysis, and each representative flow plot was repeated more than 3 times.

### Statistics.

Statistical analyses were performed in consultation with the UCLA Department of Medicine Statistic Core. For in vitro studies with cell lines, each study was repeated 3–5 times. For in vivo studies, each analysis, including those shown in bar graphs, such as the percentage, MFI comparison, or real-time PCR, contains at least 4–7 biological replicates (number of mice). For flow analysis, at least 5000 events were acquired for the population of interest. To compare statistical difference between 2 groups, Mann-Whitney *U* tests were used. For the experiment described in Figure 1C; Figure 2, A and D; and Figure 6B with multiple pairwise comparisons, Kruskal-Wallis analysis with Dunn’s test was used. All the data with error bars are presented as mean values ± SEM. All the animal data are presented as an individual mouse per dot, with median value indicated by horizontal bars. *P* values <0.05 were considered significant. Consultation on statistical analysis was performed with the UCLA Center for AIDS Research Biostatistics Core and the UCLA Biostatistics Department.

To compare statistical differences for multiple comparisons for Figure 4, B and C; Figure 5A; and Supplemental Figure 4B, LMMs with a random intercept allowed to vary by mouse were fit for all outcome variables, using restricted maximum likelihood in R (110) with the *lme4* package (111). F tests of main and interaction effects were conducted using type III sums of squares and the Kenward–Roger degrees of freedom approximation (112) with the *lmerTest* package (113). All post hoc *t* tests used the Kenward–Roger approximation, and *P* values were corrected for multiple comparisons using Tukey’s method (114). Detailed description of the LMM is provided in Supplementary Methods.

### Study approval.

PBMCs were obtained at UCLA in accordance with UCLA IRB–approved protocols under written informed consent using an IRB-approved written consent form by the UCLA Center for AIDS Research Virology Laboratory and distributed for this study without personal identifying information. Anonymized human fetal tissue was acquired through Advanced Biosciences Resources, was obtained without identifying information, and did not require IRB approval for its use. Animal research was carried out according to protocols approved by the UCLA Animal Research Committee (ARC) in accordance with all federal, state, and local guidelines. Specifically, all the experiments were carried out in accordance with the recommendations and guidelines for housing and care of laboratory animals of the NIH and the Association for the Assessment and Accreditation of Laboratory Animal Care International under UCLA ARC protocol no. ARC-2020-035.

## Author contributions

AZ and WM designed the experiments. WM, VR, HM, MAC, ST, PH, MAL, and AZ performed the experiments. WM and AZ analyzed the data. TDT performed the statistical analysis. WM and AZ wrote the manuscript with the help of SGK, OOY, and BDJ.

## Supplementary Material

Supplemental data

## Version 1 11/22/2022

Electronic publication
