# Supplementary material for: Autophagy inducer rapamycin treatment reduces IFN-I–mediated Inflammation and improves anti–HIV-1 T cell response in vivo
Source: JCI Insight. 2022 Nov 22;7(22):e159136. doi: 10.1172/jci.insight.159136 (PMC9746825; doi:10.1172/jci.insight.159136)
Supplement: Supplemental data [file jciinsight-7-159136-s156.pdf]

Supplementary Materials

Supplementary Figures

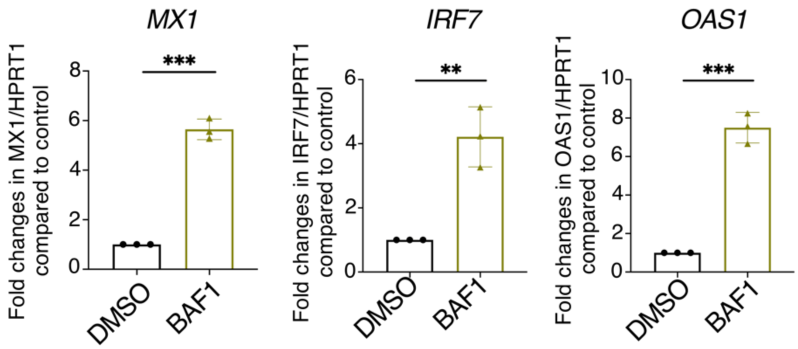

Supplement Figure 1. Autophagy inhibitor Bafilomycin A1 treatment of THP-1 cells leads to elevated expression of ISGs. THP-1 cells were treated with BafA1(50nM) for 2 days, ISGs MX1, IRF7, OAS1 and internal control HPRT1 were measured by real time PCR. Data here show the mean values of 3 independent experiments ± SEM. (represented by error bars), and $^{**}P < 0.01$, $^{***}P < 0.001$ (Mann Whitney test).

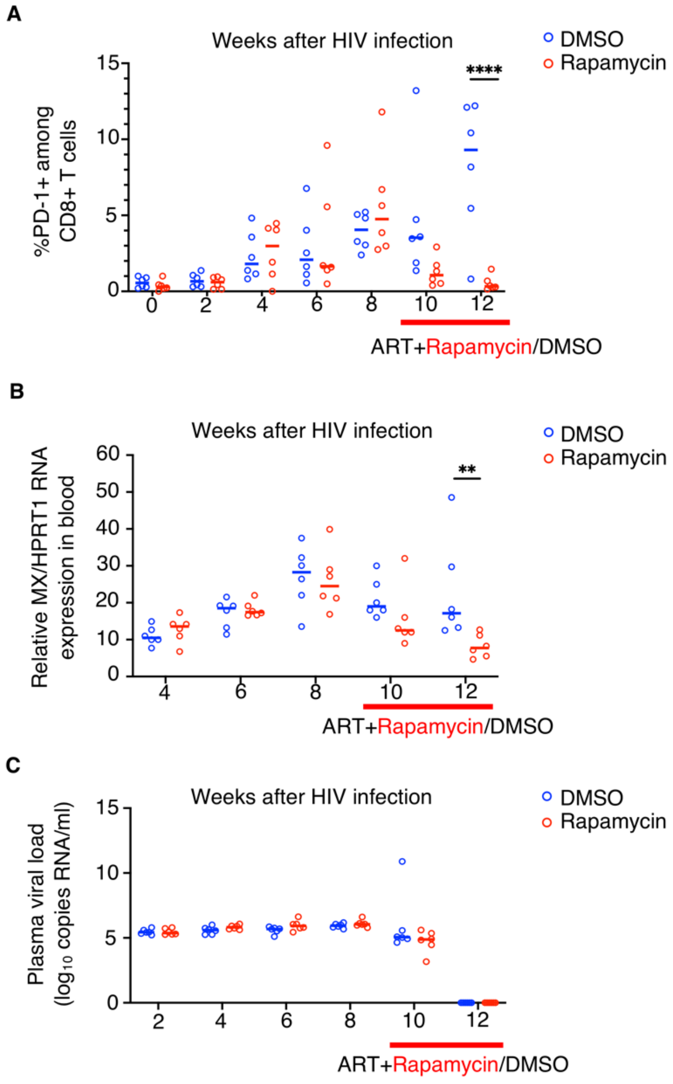

Supplement Figure 2. Longitudinal measurement of PD-1 expression, MX1 expression and viral load in humanized BLT mice that were treated with combined ART and rapamycin. As described in Figure 3, BLT humanized mice were infected with $HIV_{NFNSXL9}$ for 8 weeks. Afterwards, mice were treated with ART and Rapamycin or DMSO control for 4 weeks before necropsy. **A**) PD-1% was measured longitudinally by flow cytometry among peripheral blood CD8+ T cells from rapamycin or control mice. **B**) Expression level of MX1/HPRT1 in PBMCs from DMSO control or rapamycin treated mice as measured by real time PCR. **C**) Longitudinal measurement of viral loads in HIV-1–infected BLT mice treated with ART and DMSO or rapamycin. (n=5-7 per group) Each dot represents an individual mouse, horizontal bars indicate median values. ***P* < 0.01, *****P* < 0.0001 (Mann Whitney test).

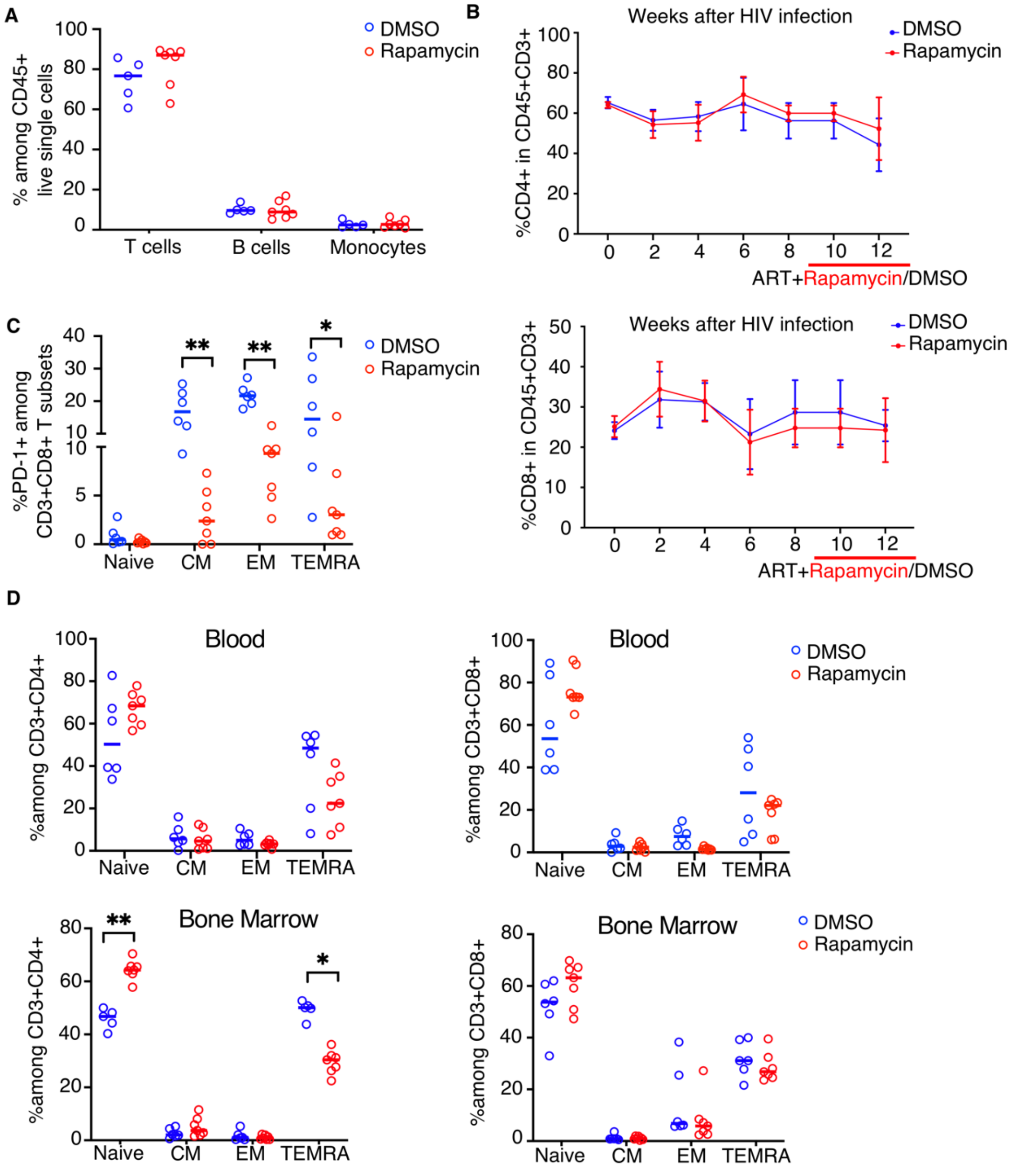

Supplement Figure 3. Lymphocytes subsets in humanized BLT mice that were treated with combined ART and DMSO or rapamycin. As described in Figure 3, BLT humanized mice were infected with $HIV_{NFNSXL9}$ for 8 weeks. Afterwards, mice were treated with ART and Rapamycin or DMSO control for 4 weeks before necropsy. **A**) Percentage of T cells (CD45+CD3+CD20-), B cells (CD45+CD20+CD3-), monocytes (CD45+CD14+CD3-CD20-) among total CD45+ in splenocytes from rapamycin or control mice. **B**) Longitudinal analysis of CD4+ and CD8+ T cells percentage among CD45+CD3+ T cells in peripheral blood from rapamycin treated or control mice. **C**) PD-1+% among peripheral blood CD8+ T cells subsets (Naïve (CD45RA+CCR7+), Central memory (CD45RA-CCR7+, CM), Effector memory (CCR7-CD45RA−, EM), Terminally differentiated effector memory T cells (CD45RA+CCR7-, TEMRA) from rapamycin or control mice. D) Frequency of Naïve, CM, EM and TEMRA cells in CD4+ (Left) or CD8+ (Right) T cells in blood and bone marrow from HIV-1–infected BLT mice treated with ART and DMSO or rapamycin. (n=5-7 per group) Each dot represents an individual mouse, horizontal bars indicate median values. *$P < 0.05$, **$P < 0.01$, Mann Whitney test.

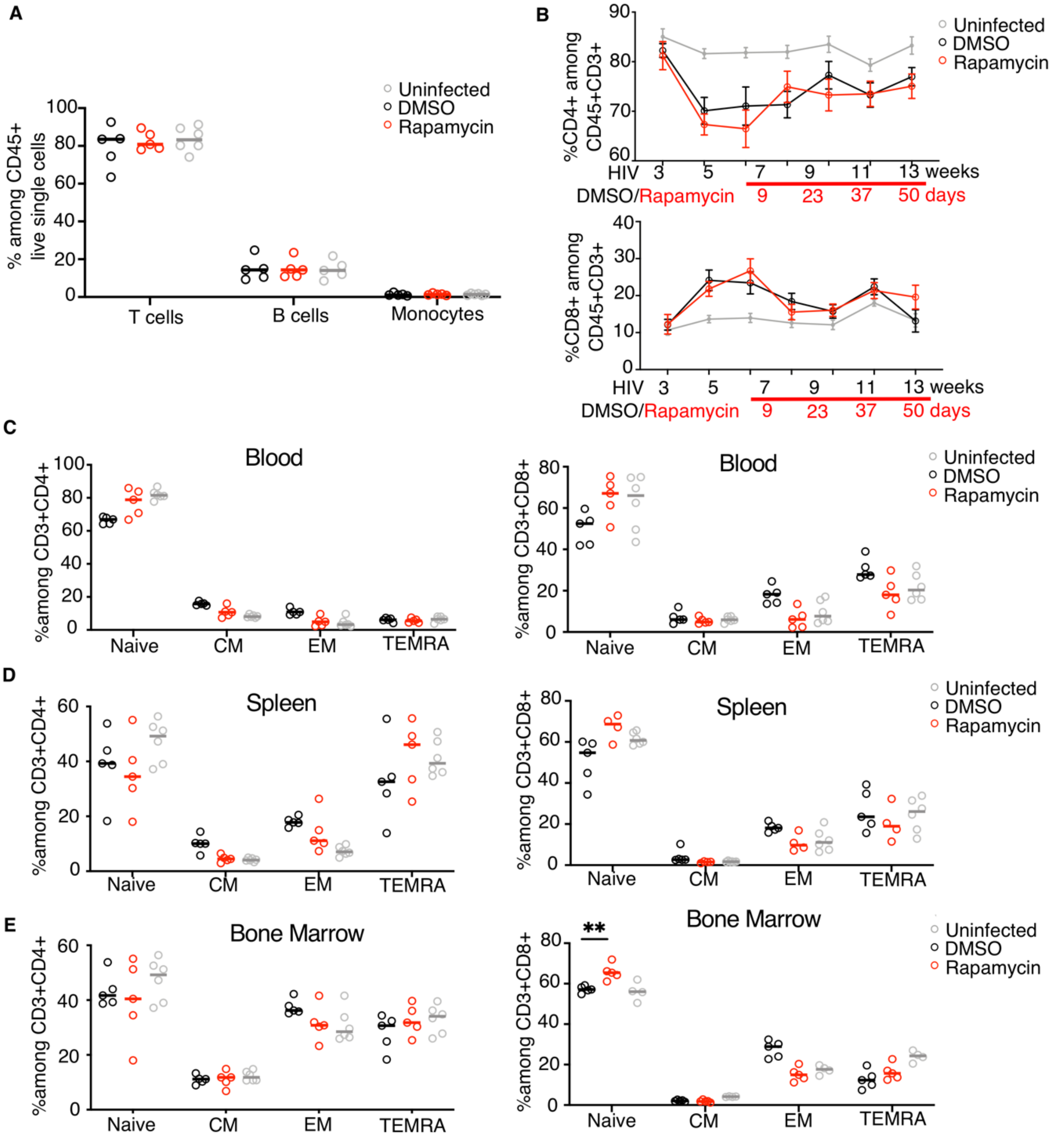

Supplement Figure 4. Differences in the percentages of lymphocytes subsets in humanized BLT mice that were prior ART-treated with rapamycin. As described in Figure 4, after HIV infection, BLT humanized mice were treated with Rapamycin or DMSO control for 2 weeks. Afterwards, while continuing rapamycin or DMSO treatment, mice were treated with ART for 3.5 weeks followed by ART interruption (ATI) for 10 days before necropsy. **A**) Percentage of T cells, B cells, and monocytes among total CD45+ in splenocytes from HIV-1 uninfected mice, or infected BLT mice that treated with rapamycin or DMSO. **B**) Longitudinal measurement of CD4+ and CD8+ T cells percentage in peripheral blood from different groups. No significance effect of rapamycin treatment was observed, tested by mixed linear model. **C-E**) Frequency of different subsets: Naïve, CM, EM, and TEMRA in total CD4 (Left) or CD8 (Right) in blood(**C**), Spleen(**D**), Bone marrow(**E**) from different treatment group. (n=4-7 per group) Each dot represents an individual mouse, horizontal bars indicate median values.

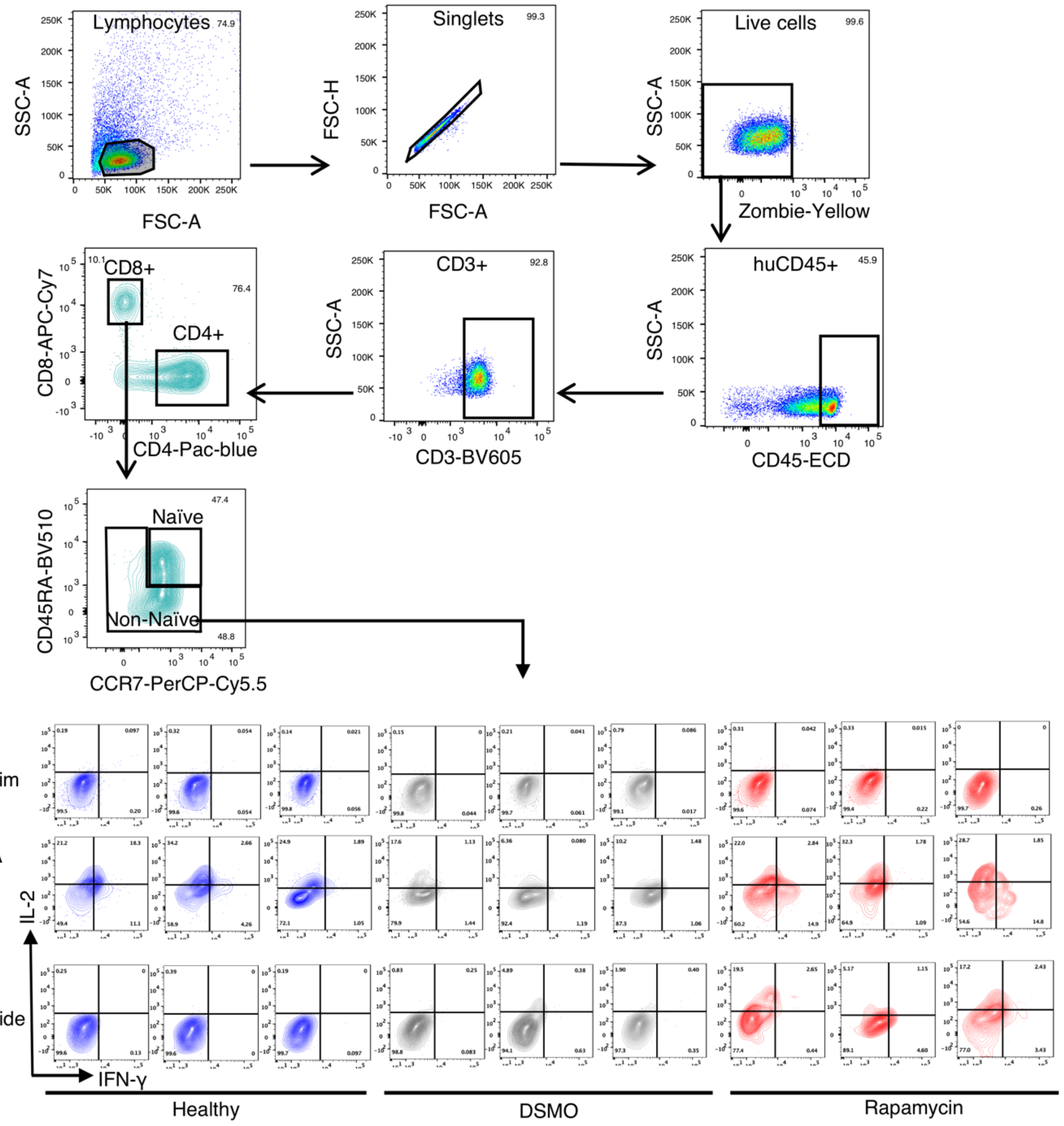

Supplement Figure 5. Representative flow cytometry plots from splenocytes illustrating the gating strategy for cytokine assay. As described in Figure 4, after HIV infection, BLT humanized mice were treated with Rapamycin or DMSO control for 2 weeks. Afterwards, while continuing rapamycin or DMSO treatment, mice were treated with ART for 3.5 weeks followed by ART interruption for 10 days before necropsy. Splenocytes were isolated at necropsy and stained with extracellular and intracellular antibodies to identify different immune cell subsets and intracellular cytokine secretion.

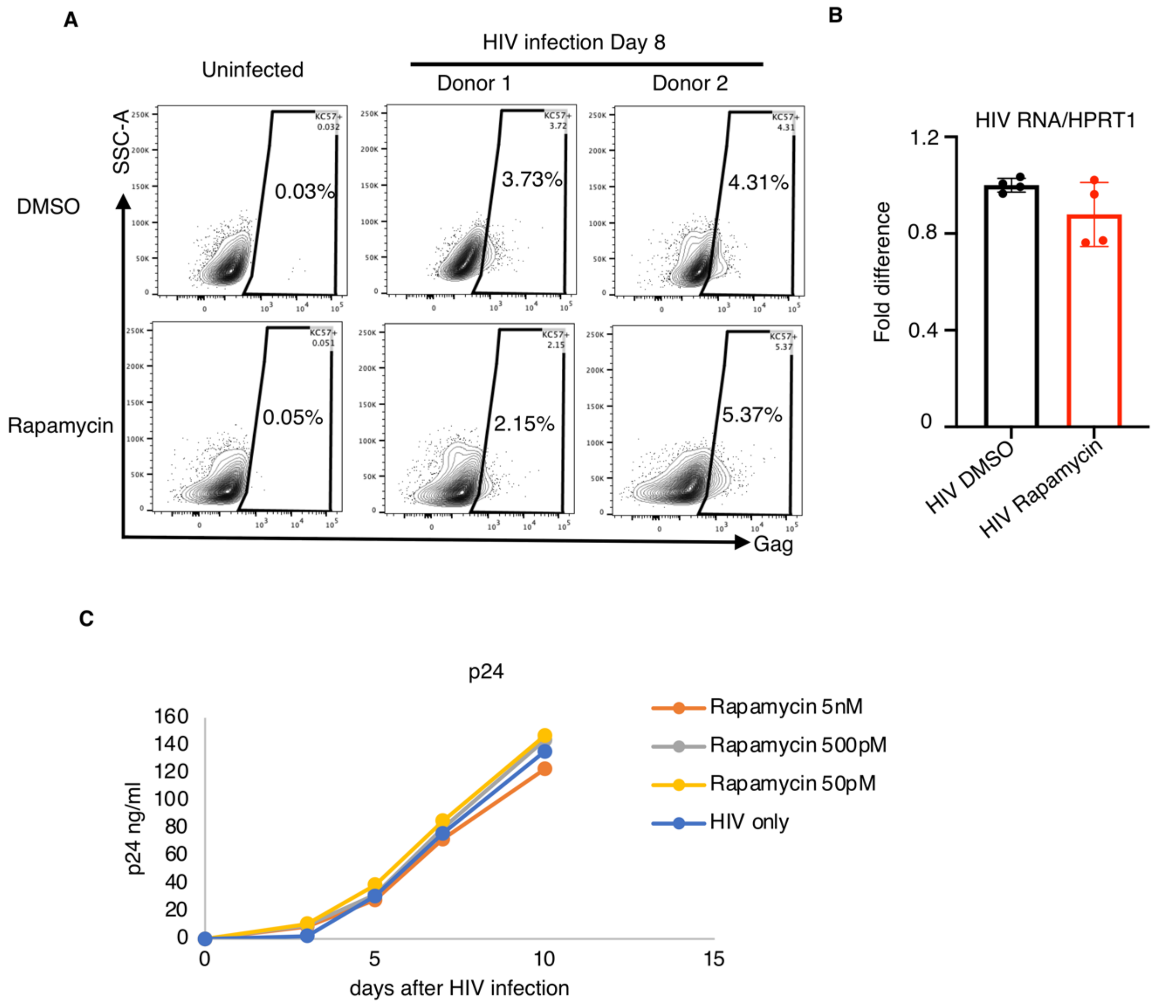

Supplementary Figure 6. Low dose rapamycin treatment does not affect HIV expression in infected primary CD4 T cells. CD14 depleted PBMCs from healthy donors were stimulated with anti-CD3/CD28 for 24 hours with DMSO or 50pM rapamycin, followed by 8 days of HIV infections in the presence of DMSO/rapamycin. **A)** Gag expression were measured by intracellular staining and flow cytometry. **B)** Real time PCR for HIV RNA and HPRT1 as an internal control. Fold difference is calculated based on HIV/HPRT1 level in DMSO treated cells. **C)** Purified CD4 cells from PBMCs were stimulated with anti-CD3/28 for with DMSO or 50pM, 500pM, 5nM rapamycin, followed by 10 days of HIV infections in the presence of DMSO/rapamycin. P24 was measured using cell supernatant collected on D0, 3, 5, 7, 10.

Supplemtary Methods

Linear mix models for Figure 4B, 4C, 5A and Supplementary Figure 4B.

Linear mixed models (LMMs) with a random intercept allowed to vary by mouse were fit for all outcome variables using restricted maximum likelihood in R Version 4.1.1 with the lme4 package. F tests of main and interaction effects were conducted using type III sums of squares and the Kenward-Roger degrees of freedom approximation with the lmerTest package (4). All post hoc t tests used the Kenward-Roger approximation as well, and p-values were corrected for multiple comparisons using Tukey's method with the lsmeans package (5).

Figure 4B: HLA-DR%, PD-1%, HLA-DR MFI, and PD-1 MFI were each entered as an outcome variable in an LMM. Two indicator variables for treatment group (one for DMSO and one for rapamycin treatment with control serving as the reference group) and one indicator variable for timepoint (one for pre with post serving as the reference group) were entered into the models as predictors. Two terms for the interaction between group and timepoint were included as predictors as well. Because residuals vs. fits plots for these models with the untransformed outcome variables indicated violations of the constant error variance assumption (i.e., the points fanned outward as the fitted values increased), the final models were run with the logarithm (base 10) of HLA-DR%, PD-1%, HLA-DR MFI, and PD-1 MFI as outcomes to stabilize the error variance.

Figure 4C: MX1, IRF7, and OAS1 were each entered as an outcome variable in their own LMM. Two indicator variables for treatment group were once again entered as predictors, as well as timepoint (treated as a continuous variable), an ART indicator variable (with no ART serving as the reference group), and two terms for the interaction between group and ART. In order to compare treatment groups after application of the treatment, we examined measurements at weeks 7, 9, 11, and 13, and the measurement taken at week 5 was entered as a covariate to control for baseline levels of the outcome. The F-tests for these initial models indicated that the interactions between group and ART were not significant ($p > .05$), so all interaction terms were dropped and the models were refit. The ART terms in these models were not significant either ($p > .05$), so another model was fit for each outcome excluding ART. Thus, the final model for each outcome contained predictors for treatment group, timepoint (treated as a continuous variable), and a baseline covariate.

Figure 5A: The logarithm (base 10) of HIV viral load was entered as an outcome variable in an LMM with the same treatment group, timepoint, ART, and group by ART interaction terms initially used for Figure 4C, with one exception: Since control group measurements of viral load were all undetectable and thus assigned a value of 1, there was no variability in the control group. Thus, control group data were excluded from the analysis, and only a single indicator variable for group (with treatment DMSO serving as the reference group) was included in the model. Once again measurements at weeks 7, 9, 11, and 13 were examined, and the logarithm (base 10) of week 5 viral load measurements was entered as a covariate.

S Figure 4B: LMMs with the same predictors initially used for Figure 4C models (treatment group, timepoint, ART, group by ART interaction, and a week 5 baseline covariate) were fit for CD4% and CD8%. As with Figure 4C, the ART interactions were not significant in either model ($p > .05$), and so the models were refit without the interaction terms. In these refit models, the ART terms were not significant either ($p > .05$), so a final model was fit for CD4% and CD8% with treatment group, timepoint (treated as continuous), and a baseline covariate entered as predictors.

References:

1. R Core Team. (2022). *R: A language and environment for statistical computing*. R Foundation for Statistical Computing. Vienna, Austria. https://www.R-project.org/
2. Bates, D., Mächler, M., Bolker, B., Walker, S. (2015). Fitting linear mixed-effects models using lme4. *Journal of Statistical Software, 67*(1), 1–48.
3. Kenward, M. G., and Roger, J. H. (1997). Small sample inference for fixed effects from restricted maximum likelihood. *Biometrics, 53*, 983–997.
4. Kuznetsova, A., Brockhoff, P. B., and Christensen, R. H. B. (2017). lmerTest package: Tests in linear mixed effects models. *Journal of Statistical Software, 82*(13), 1–26.
5. Length, R. V. (2016). Least-squares means: The R package lsmeans. *Journal of Statistical Software, 69*(1), 1-33.
